# Gut microbiota–mitochondrial inter-talk in non-alcoholic fatty liver disease

**DOI:** 10.3389/fnut.2022.934113

**Published:** 2022-09-20

**Authors:** Qi Zhang, Wenmin Xing, Qiao Wang, Zhan Tang, Yazhen Wang, Wenyan Gao

**Affiliations:** ^1^School of Pharmacy, Hangzhou Medical College, Hangzhou, China; ^2^Key Laboratory of Neuropsychiatric Drug Research of Zhejiang Province, Hangzhou Medical College, Hangzhou, China; ^3^Zhejiang Provincial Key Lab of Geriatrics, Department of Geriatrics, Zhejiang Hospital, Hangzhou, China

**Keywords:** NAFLD, gut microbiota, mitochondria, metabolites, probiotics, prebiotics

## Abstract

The increasing prevalence of non-alcoholic fatty liver disease (NAFLD), which is a progressive disease, has exerted huge a healthcare burden worldwide. New investigations have suggested that the gut microbiota closely participates in the progression of NAFLD through the gut–liver axis or gut–brain–liver axis. The composition of the microbiota can be altered by multiple factors, primarily dietary style, nutritional supplements, or exercise. Recent evidence has revealed that gut microbiota is involved in mitochondrial biogenesis and energy metabolism in the liver by regulating crucial transcription factors, enzymes, or genes. Moreover, microbiota metabolites can also affect mitochondrial oxidative stress function and swallow formation, subsequently controlling the inflammatory response and regulating the levels of inflammatory cytokines, which are the predominant regulators of NAFLD. This review focuses on the changes in the composition of the gut microbiota and metabolites as well as the cross-talk between gut microbiota and mitochondrial function. We thus aim to comprehensively explore the potential mechanisms of gut microbiota in NAFLD and potential therapeutic strategies targeting NAFLD management.

## Introduction

Non-alcoholic fatty liver disease (NAFLD) is a progressive disease initiated by an increase in hepatic lipid content, which may progress to various chronic liver diseases such as hepatic steatosis (HS), steatohepatitis, cirrhosis, and NAFLD-related hepatocellular carcinoma (HCC) (1–3). Due to its close relationship with metabolic dysfunction, NAFLD has been defined as metabolic dysfunction-associated fatty liver disease (MAFLD) (4). The prevalence of NAFLD is increasing at an alarming rate worldwide, severely affecting 20–30% of North American adults, 80% of individuals with obesity, and most patients with type 2 diabetes (T2D) (5). Remarkably, the prevalence of NAFLD may exceed 70% among children with obesity. Studies have suggested that chronic liver diseases in children, such as liver fibrosis and hepatocellular ballooning, are mainly induced by NAFLD (6). Given the overwhelming burden of NAFLD, the significant threat it poses to individuals’ health, and its poor clinical management, there is an urgent need to comprehensively illustrate its pathogenesis, develop novel non-invasive diagnostic markers, and identify potential therapeutic targets for patients with NAFLD.

**FIGURE 1:**
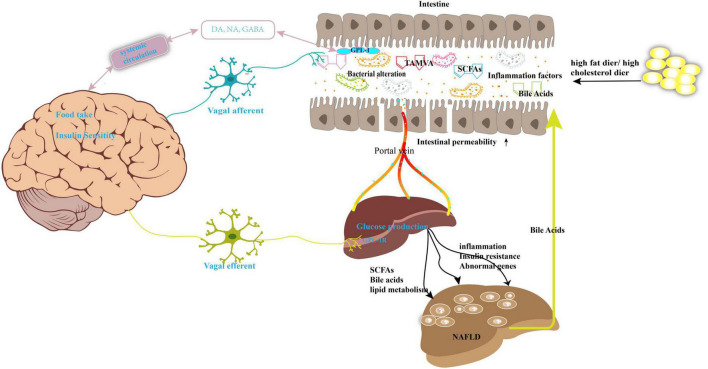
Gut microbiota imbalance contributes to the development of NAFLD. High-fat diet or high-cholesterol diet may lead the nutrition imbalance and change the gut microbiota composition and its metabolites, such as SCFAs, bile acids, and TAMVA. Simultaneously, the intestinal permeability was enhanced, and microbiota and its metabolites reach the liver through the portal vein. This abnormal biology results in dysfunction of lipid metabolism, inflammation, and then NAFLD. The gut–brain–liver axis is also involved in NAFLD. Microbiota disorders promote the intestinal endocrine L cells to secrete GLP-1 to act on the vagus nerve to activate the gut–brain–liver nerve pathway and regulate the insulin sensitivity, glucose production, fatty acid oxidation. GABA, gamma-aminobutyric acid; DA, dopamine; NA, noradrenaline; SCFAs, short-chain fatty acids; TMAVA, N,N,N-trimethyl-5-aminovaleric acid; GLP-1, glucagon-like peptide-1.

At present, a large body of evidence has revealed a strong relationship between NAFLD and imbalance of the microbiota, especially alterations in the gut microbiome (6). The gut microbiota comprises of multiple types of bacteria, fungi, viruses, archaea, and protists (7). Under normal circumstances, a balanced gut microbiota is beneficial for human health as it maintains metabolic balance of energy metabolites, lipid metabolism, and glucose metabolism (8). Moreover, the host cellular physiology and immune response are modulated by the gut microbiota (9). In contrast, imbalance of the microbiota leads to a passive increase of intestinal permeability and alteration of the homeostasis of the gut microbiota, which promotes the translocation of bacterial endotoxins or other bacterial metabolites into the systemic circulation, affecting the function of the whole body (10). The liver is the first organ exposed to the gut tract system; consequently, the liver receives the portal vein blood from the gastrointestinal tract, which contains multiple microbiota components and metabolites. However, cross-talk between the intestinal tract and the liver is mutual. For instance, the liver continuously transports bile into the small intestine through the biliary system. Consequently, the maintenance of liver homeostasis is a beneficial effect of the commensal gut microbes, whereas liver damage may result from an imbalance in the gut microbiota (11). In short, the association between the gut microbiota and NAFLD may be illustrated by the following progress (Figure 1): (1) The individual’s diet and antibiotic drugs administered during treatment affect the composition of the gut microbiota, which accelerates the development of NAFLD. (2) Metabolites produced by the microbiota, such as short-chain fatty acids (SCFAs) and bile acids (BAs), interact with the functioning of the mitochondria or genes or influence the level of inflammatory factors that promote the NAFLD process. (3) Imbalance of the gut microbiota increases intestinal epithelial barrier permeability, subsequently resulting in the influx of various substances such as harmful metabolites, lipopolysaccharide (LPS), bacteria, and bacterial DNA into the liver. (4) Serum or liver LPS levels also increase following the imbalance of the gut microbiota to evoke hepatic inflammation (12).

Furthermore, an interesting description of the “gut–brain–liver” axis, which is composed of the intestine, intestinal vagus nerve, hepatic vagus nerve, and brain, has been extensively explored to elucidate the mechanism of NAFLD pathology (13). A previous report proved that the alteration of the gut microbiota and its related bioactive metabolites may signal the processes related to the development of obesity, diabetes, and NAFLD via the gut–brain–liver cross-talk (13–16). For example, as shown in Figure 1, the gut microbiota can affect brain function and alter brain–gut peptides such as ghrelin, cholecystokinin (CCK), and glucagon-like peptide (GLP)-1 and subsequently regulate food intake and hepatic glycolipid metabolism via a negative feedback loop (17).

Recently, a close interaction between the microbiota and mitochondria has been comprehensively described in multiple diseases (18–21). Gut microbiota and their metabolites play vital roles in mitochondrial biogenesis, metabolism, and oxidative stress (18). However, the specific mechanisms by which the cross-talk between microbiota and mitochondria contributes to the progression of NAFLD are poorly understood. In this review, we elucidate the role of the gut microbiome and the metabolites of the microbiota in NAFLD based on published evidence between 2018 and 2022; we focused on the changes in the composition of the microbiota and metabolite as well as the fascinating inter-talk between the gut microbiota and mitochondria. Moreover, the potential effects are discussed in the context of exploring novel therapeutic strategies to alter the intestinal microbiome for the treatment of NAFLD.

## Link of microbiota to the non-alcoholic fatty liver disease via gut–liver axis

### Changes in the composition of the gut microbiota

Gut microbiota consists of multiple microbes, such as bacteria, fungi, archaea, and viruses. Bacteroidetes and Firmicutes are the dominant phyla among the gut bacterial microbiome (22). In general, the gut microbiota plays several pivotal roles in maintaining a healthy homeostasis, including preventing pathogen colonization, metabolizing xenobiotics, and producing vitamins, particularly those involved in energy regulation and maintaining a mature immune system such as folate and biotin (23). Subsequently, unbalanced gut microbiota may promote the occurrence and development of multiple diseases. Recently, focus has increased on the intestinal bacteria related to liver diseases, including hepatitis, cirrhosis, and NAFLD (Table 1). “Dysbiosis” has been used to illustrate the changes in the composition of the gut microbiota and is also characterized as the imbalance or alteration of the microbiota in a way that can be harmful to the host. Indeed, the composition of the gut microbiota shows marked dynamic changes from birth to adulthood and old age (24). However, most alterations in the gut microbiota are closely related to environmental influences such as sex, diet, and drugs administered during treatment (25).

**TABLE 1:** Investigations into NAFLD and microbiota composition and function in humans.

Study	Design and participant details	Microbial findings and summary of results	Upregulated microbiota	Downregulated microbiota
Caussy et al. (63)	• Prospective discovery cohort • 203 participants were divided into NAFLD- cirrhosis, NAFLD without AF, non-NAFLD controls	Identified a specific stool microbiome-derived signature of NAFLD-cirrhosis Assessed microbial biomarker could present an adjunct tool to determine stage of liver disease.	Streptococcus, Megasphaera, Enterobacteriaceae, Streptococcus, Gallibacterium	Bacillus and Lactococcus, Pseudomonas, *Faecalibacterium prausnitzii*, Catenibacterium, Rikenellaceae, Mogibacterium, Peptostreptococcaceae
Behary et al. (39)	• Prospective discovery cohort • 90 subjects were divided into: 32 with NAFLD-HCC, 28 with NAFLD-cirrhosis and 30 non-NAFLD control	Gut dysbiosis characterizes liver Cirrhosis NAFLD-HCC was characterized by expansion of Proteobacteria compared to non-NAFLD controls.	Proteobacteria, Coriobacteriaceae	Oscillospiraceae, Erysipelotrichaceae, Bacteroidetes, Muribaculaceae, Odoribacteraceae, Prevotellaceae
López-Salazar et al. (109)	• Animal model • SO, OO, CO was fed with NAFLD C57BL/6 mice, respectively	SO showed the highest microbial diversity, high insulin sensitivity and low grade inflammation. CO showed the lowest bacterial diversity, increase in the LPS concentration, hepatic steatosis, increased lipogenesis.	*Akkermansia muciniphila*, Bifidobacterium, *Bacteroides acidifaciens*, *Faecalibacterium prausnitzii*, *Mucispirillum schaedleri*	Verrucomicrobia phyla
Ding et al. (26)	• Animal model • Mice were randomized into: CHOW, CHOW + NAC, HFD + NAC	NAC potentially alleviated HFD-induced NAFLD via the homeostasis of the gut microbiota.	Firmicutes norank_f_Erysipelotrichaceae, Coriobacteriaceae_UCG-002	Bacteroidetes, Enterorhabdus, Lachnoclostridium, Alistipes
Liu et al. (50)	• Prospective discovery cohort • Female NAFLD patients and normal controls. • Female C57BL/6 mice were divided into: normal diet, sham-operated + HFD, OVX + HFD, OVX + HFD + FMT.	The structure of the gut microbiota was changed in NAFLD patients and mice induced by OVX. FMT attenuated estrogen deficiency induced NAFLD in mice.	Bacteroidetes, Proteobacteria, Bacteroides, Alistipes, Verrucomicrobia, Faecalibaculum, Helicobacter, Epsilonbacteraeota	Muribaculaceae, Lactobacillus
Zeybel et al. (4)	• Prospective discovery cohort • MAFLD were classified into: no steatosis, mild steatosis, moderate steatosis, severe steatosis.	The alterations in the microbial compositions start at early stages of the clinical spectrum and cause metabolic disturbances underlying HS.	Firmicutes (*Streptococcus mitis* and *Roseburia inulinivorans*) and Bacteroidetes (*Barnesiella intestinihominis* and *Bacteroides uniformis*)	Bacteroidetes (Prevotella sp. CAG 520, Prevotella sp. AM42 24, *Butyricimonas virosa*, and *Odoribacter splanchnicus*), Proteobacteria (*Escherichia coli*), Lentisphaerae (*Victivallis vadensis*), and Firmicutes (*Holdemanella biformis*, *Dorea longicatena*, *Allisonella histaminiformans*, and *Blautia obeum*)
Zhang et al. (38)	• Animal model • Male C57BL/6 were fed with NC, HFLC, HFHC	High dietary cholesterol induces spontaneous and progressive development of NAFLD–HCC in male mice by modulating the gut microbiota.	M. schaedleri_Otu038, Desulfovibrio_Otu047, Anaerotruncus_Otu107, Desulfovibrionaceae_Otu073	Akkermansia, Lactobacillus, Bifidobacterium, Bacteroides
Carbajo-Pescador et al. (56)	• Wistar rats were separated into: Control, semi-purified high fat diet (HFD)	The consumption of oils with high monounsaturated and polyunsaturated fats and probably the presence of phenolic compounds that protects gut barrier integrity allows the maintenance of healthy gut microbiota.	Firmicutes Clostridia (Firmicutes phylum), Deltaproteobacteria and Gammaproteobacteria (Proteobacteria phylum)	Bacteroidetes, Bacteroidia (Bacteroidetes phylum) and Bacilli (Firmicutes phylum)
Zhang et al. (84)	• C57BL/6J male mice were divided into: normal diet, HFD, HFID (high-fat plus resistant dextrin diet)	Resistant dextrin mitigates hepatic steatosis through modifying the intestinal microbiome and fecal metabolome in mice.	Bifidobacteriaceae, Dietziaceae, and Prevotellaceae, Firmicutes phylum, Turicibacter, Faecalibaculum, and Streptococcus	Bifidobacterium, Dietzia, Globicatella, Enterococcus, Lactobacillus, Leuconostoc, Lactococcus, Streptococcus, Lachnoclostridium, Parabacteroides, Catabacter, Blautia, Dubosiella, Erysipelatoclostridium, unidentified_ Erysipelotrichaceae
Li et al. (28)	• Male C57BL/6 mice were divided into: normal diet, HFHC diet, HFHC diet supplemented with UCDA.	UDCA could resistance hepatic inflammation in a dose dependent pattern and improve the dysbiosis of the gut microbiota induced by HFHC.	Firmicutes, Verrucomicrobiota	Bacteroidetes, Actinobacteriota, Proteobacteria

SO, soybean oil; OO, olive oil; CO, coconut oil; NAC, N-acetylcysteine; CHOW, chow diet; HFD, high-fat diet; NC, normal chow; HFLC, high-fat/low-cholesterol diet; HFHC, high-fat/high-cholesterol diet; OVX, ovariectomy.

#### High-fat diet-induced changes in the microbiota in non-alcoholic fatty liver disease

We have summarized recent studies on the alteration of the composition of the microbiota in the development of NAFLD through the gut–liver axis (Table 1). For example, NAFLD is commonly induced by nutritional imbalance, which results from dietary disorder-induced overnutrition and malnutrition. Moreover, comprehensive examination of the NAFLD etiology induced by overnutrition and obesity showed that an alteration of the gut microbiota has emerged as a crucial element in promoting the occurrence of NAFLD (6). The levels of alanine aminotransferase (ALT), aspartate aminotransferase (AST), and triglycerides (TG) were significantly elevated by a high-fat diet (HFD) in C57BL/6 mice with NAFLD (26). Consequently, HFD decreased the special operational taxonomic units (OTUs) and Shannon diversity index of the microbiota, which suggested that HFD caused an imbalance in the homeostasis of the gut microbial community. HFD remarkably enhanced the abundance of Firmicutes and reduced the abundance of Bacteroidetes. Moreover, participants with obesity exhibited a significantly higher ratio of Firmicutes to Bacteroidetes (27). In another study, Li et al. (28) found that the high-fat high-cholesterol (HFHC) group upregulated the abundance of Firmicutes and *Verrucomicrobiota* and downregulated the abundance of *Bacteroidetes*, *Actinobacteria*, and *Proteobacteria*. However, a gradual decrease in the abundance of *Firmicutes*, *Verrucomicrobiota*, and *Actinobacteriota* and a gradual increase in the abundance of Bacteroidetes were identified during the progression of NAFLD from non-alcoholic steatohepatitis (NASH) to NASH with fibrosis. In fact, the levels of TG and total cholesterol (TC) in the liver were strongly correlated with the abundance of Firmicutes and Bacteroidetes in HFD-induced NAFLD mice. Moreover, the alteration of serum lipid levels was also related to imbalanced bacterial microbiota, including *Erysipelotrichaceae*, *Coriobacteriaceae*, *Enterorhabdus*, *Lachnoclostridium*, and *Alistipes* in C57BL/6J mice fed with HFD. Furthermore, the gut microbiome profile differs according to the severity of NAFLD. A previous cross-sectional analysis that included NAFLD-cirrhosis, NAFLD without advanced-fibrosis, and non-NAFLD controls investigated alterations in the composition of the microbiota (29). The β-diversity of the gut microbiota was lower among patients with NAFLD without advanced fibrosis than among participants of the healthy control group, whereas it was higher among patients with NAFLD-cirrhosis than in patients with NAFLD without advanced-fibrosis. Consequently, a decrease in gut microbiota diversity was identified in proportion to NAFLD severity. Furthermore, *Streptococcus* abundance increased in patients with both NAFLD-cirrhosis and NAFLD without advanced fibrosis; *Megasphaera* abundance only increased in participants with NAFLD-cirrhosis. However, Bacillus and Lactococcus abundance increased in patients with NAFLD without advanced fibrosis and in healthy participants. Meantime, the abundance of *Enterobacteriaceae*, *Streptococcus*, and *Gallibacterium* was enhanced in patients with NAFLD-cirrhosis, while *Faecalibacterium prausnitzii*, *Catenibacterium*, *Rikenellaceae*, *Mogibacterium*, and *Peptostreptococcaceae* were only identified in healthy participants. The composition of the gut microbiome also significantly differed with different severities of HS (4). The abundance of *Bacteroidetes*, *Proteobacteria*, *Lentisphaerae*, and *Firmicutes* was largely decreased in patients with mild steatosis. In contrast, the abundance of *Firmicutes* and *Bacteroidetes* was significantly increased in patients with moderate steatosis. The abundance of *Actinobacteria*, *Bacteroidetes*, *Lentisphaerae*, *Firmicutes*, and *Proteobacteria* was also notably decreased in patients with severe steatosis. The abundance of the Firmicutes bacterium CAG 95 was also significantly decreased in patients with both severe and moderate steatosis. Similar to previous studies, some species of the phylum *Firmicutes*, including *Ruminococcus bromii*, *Dorea longicatena*, and *Roseburia* sp. CAG 182, could regulate AST, ALT, and uric acid levels.

#### Sex-dependent gut microbial features related to non-alcoholic fatty liver disease

Sex hormones and sex chromosomes are the two major factors driving sex-based characterization of the differences in the microbiome between the male and female sexes (30). A previous study revealed that sex-specific microbiomes may play an essential role in the incidence of NAFLD and obesity (31). For instance, the genus *Holdemanella* and family *Erysipelotrichaceae* were negatively related to the android fat ratio in females, whereas a positive relationship was identified in males. Meanwhile, the family *Ruminococcaceae* was positively related to the gynoid fat ratio only in females. Male and female sexes have different microbiome species associated with fat distribution, and sometimes, the same family and genus of microbiomes have different associations with fat distribution in the two sexes (32). Postmenopausal females with estrogen deficiency display a higher risk for NAFLD progression to fibrosis owing to the alteration of gut microflora (30). Male patients with NAFLD showed a decreasing trend in microbial α-diversity, an increasing trend in the abundance of genera *Dialister*, *Streptococcus*, and *Bifidobacterium* species, and a decreasing trend in the abundance of the genera *Phascolarctobacterium*, *Mogibacteriaceae*, *Rikenellaceae*, and *Peptococcaceae*. In contrast, female patients with NAFLD had an increasing trend in microbial α-diversity and the abundance of these taxa and showed an opposite trend (33). As previously described, *Dialister* is a genus of Firmicutes that increases in abundance in patients with liver cirrhosis (3). The genus *Phascolarctobacterium* showed association with control of the body weight of patients with NAFLD (34). RF39 elicits a potential health benefit in controlling BMI, blood TG, and frailty among older adults (35). The specific changes in microbiota induced by different maternal diets were also notable. For example, the abundance of *Firmicutes* and *Tenericutes* was increased and that of *Bacteroidetes*, *Verrucomicrobia*, and *Cyanobacteria* was decreased among male mouse offspring due to HFD. Female mouse offspring had a higher abundance of *Firmicutes*, *Saccharibacteria*, and *Deferribacteres* and lower *Bacteroidetes* and *Verrucomicrobia* in the HFD group than in the control group (36). However, the mechanism underlying the alteration of microbiota induced by differences in sex remains poorly explored.

#### The alteration of microbiota in non-alcoholic fatty liver disease-hepatocellular carcinoma

Non-alcoholic fatty liver disease is one of the main factors contributing to HCC (37). The prevalence of NAFLD-HCC has increased more than that of hepatitis, and the frequency of liver transplantations is rapidly growing worldwide. The gut microbiome is a crucial factor that promotes the occurrence of NAFLD and NAFLD-HCC (3, 38, 39). NAFLD-HCC is characterized by an increased abundance of *Proteobacteria* compared to that in healthy individuals. An increase in *Enterobacteriaceae* and decrease in *Oscillospiraceae* and *Erysipelotrichaceae* abundances were identified in patients with NAFLD-HCC. However, the microbiome signature differed between patients with NAFLD-cirrhosis and NAFLD-HCC. An increase in *Eubacteriaceae* abundance was observed in NAFLD-cirrhosis group, which was not found in either NAFLD-HCC or the non-NAFLD control groups. Furthermore, an elevated abundance of *Coriobacteriaceae* and a lower abundance of *Muribaculaceae*, *Odoribacteraceae*, and *Prevotellaceae* were also detected in those with NAFLD-cirrhosis (3, 39). Moreover, supplementation of the diet with cholesterol spontaneously promoted the occurrence of NAFLD-HCC, followed by dysbiosis of the gut microbiota. This report confirmed that an increase in *Helicobacter ganmanii* and a decrease in *Bacteroides* play an essential role in NAFLD-HCC onset. In contrast, the imbalanced gut microbiota regulates cholesterol levels in NAFLD-HCC. For example, some bacteria such as *Mucispirillum schaedleri*, *Desulfovibrio*, *Anaerotruncus*, and *Clostridium celatum* were positively correlated with cholesterol levels. However, *Bifidobacterium*, *Bacteroides acidifaciens*, *Bacteroides uniformis*, *Akkermansia muciniphila*, and *Lactobacillus* were negatively correlated with serum and liver cholesterol levels. Similar results were also observed in cases of hypercholesterolemia; *Bifidobacterium* and *Bacteroides* negatively regulate the levels of TC and low-density lipoprotein (LDL) cholesterol in the serum (38).

Notably, the abundance of bacterial species is closely associated with host gene expression in NAFLD. First, *Barnesiella*, *Oscillibacter* sp. CAG 241, and *Roseburia* related to HS could regulate the expression of inflammatory genes. For example, *Campylobacter concisus* and *Porphyromonas endodontalis* negatively regulated the expression of CXCL9 and LIF-R, respectively, and *Veillonella atypica* positively regulated the expression of CD244. Based on these data, we can infer that these inflammation-related proteins are responsible for enhancing antigen presentation to lymphocytes, which break the liver immune tolerance and stimulate both cellular and humoral immune responses in NAFLD (4, 39). In addition, some species of bacteria, including *Bacteroides caecimuris*, *Bacteroides xylanisolvens*, and *Clostridium bolteae*, were able to regulate the levels of IL-10+ Tregs and CD8+ T-cells in patients with NAFLD-HCC, suggesting that these bacteria participate in the modulation of adaptive immunity (39). Second, members of the predominant bacterial phyla, *Firmicutes* and *Bacteroidetes*, also influence the lipid metabolism pathway in the liver by regulating the related genes. Firmicutes positively regulate the expression of Mogat1 and CD36 in the liver. In contrast, Bacteroidetes negatively regulate the expression of Cidea, CD36, Acnat2, Mogat1, and GPAT3. In addition, *Erysipelotrichaceae* positively regulated the expression of Cidea, CD36, Acnat2, Mogat1, and GPAT3. Previous studies have suggested that Cidea, CD36, and GPAT3 are involved in the lipid metabolism pathway that drives the occurrence of HS (26). Third, *F. prausnitzii* can also regulate genes related to other pathways, such as IRS-1/2, IL-6, SOCS3, LEPR, and steroid response element binding protein-α (SREBP-α), to prevent the development of NAFLD (40). In addition, *F. prausnitzii* regulates the immune response by mediating the expression of PRKCZ, STAT3, and IRS2. Another bacterium, *Ruminococcus* spp., has also been found to regulate the expression of AKR1B10, which is related to apoptosis (41). The abundance of *Ruminococcus* spp. is also positively related to JUN and JUNB, which attenuates the pathogenesis of NASH (42).

### Changes in the metabolites of the gut microbiota due to non-alcoholic fatty liver disease

Metabolites in the circumstance system were also altered, following the changes in the gut microbiota, which highlighted the etiological mechanisms of NAFLD. Data are available describing specific metabolite signatures related to the different stages of NAFLD (4). Furthermore, gut bacterial metabolites participate in maintaining homeostasis and affect the development of NAFLD through the gut microbiota–liver axis (Table 2). The following descriptions focus on the foremost metabolites of the gut microbiota recently described to be involved in NAFLD progression.

**TABLE 2:** Investigations into NAFLD and microbiota metabolites.

Study	Design and participant details	Microbial findings and summary of results
Caussy et al. (63)	• Prospective cohort study • 156 well-characterized Twins and Families with NAFLD	NAFLD individuals had a microbial origin and derive potentially from the gut microbiome: 3-(4-hydroxyphenyl) lactate, N-formylmethionine, phenyllactate, mannitol, allantoine, N-(2-furoyl)glycine.
Zhao et al. (43)	• Prospective cohort study • 15 patients with liver steatosis and 15 controls • 1157 subjects with liver steatosis 766 subjects control	The intestinal bacteria *Enterococcus faecalis* and *Pseudomonas aeruginosa* to metabolize trimethyllysine to TMAVA. TMAVA to bind and inhibit BBOX, reducing synthesis of carnitine.
Behary et al. (39)	• Prospective discovery cohort • 90 subjects were divided into: 32 with NAFLD-HCC, 28 with NAFLD-cirrhosis and 30 non-NAFLD control	Isocitrate was lower in NAFLD-HCC subjects The feces of NAFLD-HCC subjects were enriched in acetate, Oxaloacetate, acetylphosphate, butyrate, and formate, propionate, malonate.
Liu et al. (50)	• Prospective discovery cohort • Female NAFLD patients and normal controls. • Female C57BL/6 mice were divided into: normal diet, sham-operated + HFD, OVX + HFD, OVX + HFD + FMT.	Peptococcus and Romboutsia were positively, while Ruminiclostridiun-6 and Muribaculum were negatively correlated with SCFA. The butyrate content was much lower in the NAFLD patients. OH group had a significantly greater expression of the lipid intake related gene VLDLR, and lower expression of the lipid oxidation related genes, PPAR-α and ACAA.
Zeybel et al. (4)	• Prospective discovery cohort • NAFLD were classified into: no steatosis, mild steatosis, moderate steatosis, severe steatosis.	TMAVA plasma level was significantly increased in moderate steatosis vs. no steatosis
Zhang et al. (38)	• Animal model • Male C57BL/6 were fed with NC, HFLC, HFHC	Cholesterol induces increased TCA and decreased IPA through gut microbiota alteration, thereby promoting lipid accumulation, cell proliferation in the liver, leading to NAFLD–HCC development.
Carbajo-Pescador et al. (56)	• Wistar rats were separated into: Control, semi-purified high fat diet	Exercise increased SCFAs production in the early obesity and NAFLD model. Exercise improves HFD-mediated barrier disruption and counteracts endotoxemia, oxidative stress, gut-liver axis activation and inflammatory response in our *in vivo* model of early obesity and NAFLD. Exercise improves HFD-induced obesity and hepatic steatosis through its capacity to modulate bile acids metabolism and enterohepatic circulation.
Zhang et al. (84)	• C57BL/6J male mice were divided into: normal diet, HFD, HFID (high-fat plus resistant dextrin diet)	HFID-fed increased the levels of lactitol, maltitol, trigonelline, carvone, and dehydroepiandrosterone, decreased testosterone, lipoic acid, oleic acid, and tryptophan.

TMAVA, N,N,N-trimethyl-5-aminovaleric acid; TCA, taurocholic acid; IPA, 3-indolepropionic acid; SCFAs, short-chain fatty acids; NAC, N-acetylcysteine, CHOW, chow diet; HFD, high-fat diet; NC, normal chow; HFLC, high-fat/low-cholesterol diet; HFHC, high-fat/high-cholesterol diet; OVX, ovariectomy; OH, sham-operated + high-fat diet.

#### N,N,N-trimethyl-5-aminovaleric acid

N,N,N-trimethyl-5-aminovaleric acid (TMAVA) is a novel metabolite identified in patients with HS which is useful for characterizing the different severities of HS (43). Zhao et al. (43) found that plasma trimethyl lysine (TML) is a precursor of TMAVA. *Enterococcus faecalis* and *Pseudomonas aeruginosa* can promote TML metabolism into TMAVA (44). Interestingly, increased levels of TML were observed in patients with steatosis. In another clinical trial (4), the plasma level of TMAVA was positively dependent on the abundance of *Bacteroides stercoris*, *B. uniformis*, and *Parabacteroides distasonis* and negatively dependent on the abundance of *Prevotella copri*. However, TMAVA was significantly decreased by the combined metabolic activators (CMAs). In contrast, TMAVA can bind and inhibit the expression of γ-butyrobetaine hydroxylase (BBOX) to decrease carnitine synthesis (45). Thus, TMAVA participates in energy production and conversion and the metabolism and transport of carbohydrates and lipids in the liver. Therefore, TMAVA can be considered a potential metabolite signature for the prediction of NAFLD.

#### Short-chain fatty acids

Short-chain fatty acids are a primary type of bacterial metabolite produced by bacterial fermentation of otherwise indigestible fibers in the colon (46). Many previous studies have illustrated the role of aberrant levels of SCFAs in NAFLD progression (39, 40, 47–50). SCFAs can disrupt the functioning of the intestinal barrier impairing the translocation of LPS and increasing the level of liver endotoxemia to promote the pathogenesis of NAFLD (50). Butyrate and propionate are the main components of SCFAs, which can decrease gut inflammation and improve gut barrier integrity to limit LPS translocation (51). Liu et al. (50) confirmed that butyrate was significantly reduced in female patients with NAFLD and in ovariectomized (OVX) mice. Butyrate was also positively correlated with Tregs and effector IL-10 and negatively correlated with cytotoxic CD8 T-cells in participants with NAFLD-HCC (39). Indeed, previous studies have indicated that gut microbiota directly regulates T-cell immunity through SCFAs (52, 53). Moreover, supplementation of a high fiber diet increases the levels of SCFAs, especially butyrate, to promote hepatocyte proliferation (49, 54). Butyrate, nicotinate, and 2-oxoglutarate positively regulate hepatic oxidative phosphorylation and negatively regulate TG content through oxidative metabolism. The intermediates of SCFAs, oxaloacetate and acetylphosphate, were also increased in patients with NAFLD-HCC (39).

Short-chain fatty acids are closely related to specific bacterial species. For example, *F. prausnitzii* can produce SCFAs to induce apoptosis by regulating mitochondrial death, reactive oxygen species (ROS), and the caspase pathway during the progression of NAFLD to NASH (55). SCFA levels were also positively dependent on *Peptococcus* and *Romboutsia* and negatively dependent on *Ruminiclostridiun-6* and *Muribaculum* (40). SCFAs and these bacteria were found to positively regulate the levels of TC, leptin, and body weight in female participants. In addition, *Olivibacter*, *Clostridium*, and *Dysgonomonas* may be related to levels of other SCFAs, such as acetate and propionate (56).

#### Bile acid

In addition to SCFAs, BA can also regulate inflammation due to HS by agonizing or antagonizing their cognate receptors (38, 51, 57). Primary BAs, such as taurocholic acid (TCA), tauroursodeoxycholic acid (TUDCA), glycocholic acid (GCA), and taurochenodeoxycholic acid (TCDCA), were aberrantly elevated in patients with NASH and mice fed a HFHC diet. Indeed, it had been confirmed that TCA, GCA, TCDCA, and TUDCA are critical metabolites that affect the accumulation of hepatic lipids and inflammation. Xiang et al. (38) reported that an increase in the abundance of *M. schaedleri*, *Roseburia*, and *H. ganmanii* possibly elevated TUDCA, TCDCA, TCA, and GCA levels, whereas decreased abundance of *A. muciniphila* due to HFHC diet elevated TCDCA and TUDCA levels. Increased abundance of *Anaerotruncus* due to a HFHC diet depleted the level of indolepropionic acid (IPA) (57). In addition, other bacteria such as *Roseburia intestinalis*, *P. distasonis*, *Bacteroides vulgatus*, and *B. uniformis* are also involved in the secondary BA metabolism pathway (4). In participants with NAFLD, primary BA levels were negatively related to the abundance of *R. bromii*, a species beneficial to human health. In addition, the enrichment of *Bilophila wadsworthia* lead to BA dysmetabolism, inflammation, and intestinal barrier dysfunction in the host, inducing higher glucose dysmetabolism and HS (58). Thus, *Bifidobacterium* and *Bacteroides*, the predominant bacteria of the gut microbiota, also participate in BA metabolism in NAFLD induced by HFHC feed (38). These species could prevent the transformation of taurine- and glycine-conjugated BAs into their unconjugated free forms (59). Collectively, previous findings suggest that treatment strategy exploration for NAFLD may be realized by reversing impaired BA metabolism, thereby preventing the development of NAFLD-HCC.

#### Other metabolites

Fatty acid (FA), which may be produced as a result of metabolism by *Firmicutes* bacterium CAG 95 and *Firmicutes* bacterium CAG 110, is also involved in the development of NAFLD (4). The expression of crucial hepatic genes, including SREBP1, PPAR-γ, FAS, and CHREB, is involved in FA synthesis in HFD-fed mice and patients with estrogen reduction (56). Lipid accumulation in the liver is partly responsible for the uptake of circulating FA and decreases in the rate of FA oxidation and secretion (60). Indeed, butyrate can reverse PPAR-α activation to enhance FA β-oxidation, inhibit lipid synthesis, and deplete the level of nuclear factor-kappa B (61, 62), which was also observed in NAFLD-OVX mice (50).

In addition, 3-(4-hydroxyphenyl) lactate is a newly defined amino acid involved in tyrosine metabolism in NAFLD (63). Interestingly, circulating 3-(4-hydroxyphenyl) lactate may be generated by *Escherichia coli*, which also produces hydroxyphenyllactate *in vitro* (64). Moreover, members of *Firmicutes*, *Bacteroidetes*, and *Proteobacteria* phyla can also produce 3-(4-hydroxyphenyllactacte and phenyllactate) in NAFLD. Furthermore, many other dysfunctional metabolites are related to bacterial abundance. For example, carnosine, nicotinate, methylamine, trimethylamine, and arabinose were associated with the abundance of *Bacteroides* in HFD-induced NAFLD. *Olivibacter*, *Clostridium*, and *Dysgonomonas* have been correlated with acetate and propionate (56).

## Link between microbiota and non-alcoholic fatty liver disease via gut–brain–liver axis

The gut, brain, and liver interact closely with each other. For example, intestinal signals can activate the hypothalamic lipid-sensitive signals via the vagal afferent nerves, which in turn controls food intake (65). Simultaneously, the brain inhibits hepatic glucose production to suppress the onset of obesity. In turn, the liver inhibits hepatic glucose output via the insulin signaling pathway to reduce brain glucose uptake and the impairment of neuronal cell activity (66). On the one hand, gut dysbiosis induced by high-fat/high-sugar diet increases intestinal permeability and promotes the production of inflammatory cytokines in colonic epithelial cells (13). As a result, the vagal gut–brain communication is altered. The gut-vagal afferent nerve is continuously activated due to inflammation, which signals a response in the brain to induce a series of inflammation-associated sickness-causing behaviors in the liver, such as insulin sensitivity and HS (67). On the other hand, GLP-1 and GLP-1R play a predominant role in the gut–brain–liver axis by predominantly promoting insulin secretion in a glucose-dependent manner and reducing the body weight through a variety of channels (68). Importantly, the gut microbiota is closely related to GLP-1 secretion during NAFLD development. Studies have suggested that gut microbial dysbiosis and its related metabolites can stimulate GLP-1 secretion through the GPR41/43 pathway and ultimately lead to the accumulation of fat, which leads to NAFLD (69).

In addition, SCFAs, the main metabolites of the gut flora, can simulate vagus nerve signaling and regulate the levels of neurochemicals, such as serotonin, dopamine (DA), and noradrenaline to influence brain function (70). Moreover, SCFAs are also responsible for the modulation of the host’s appetite and food intake to promote the release of GLP-1 and peptide YY (via interaction with G-coupled proteins expressed by enteroendocrine cells to activate the gut–brain–liver axis) (71). This contributes to the occurrence of NAFLD.

## Gut microbiome signaling to mitochondria in non-alcoholic fatty liver disease

**FIGURE 2:**
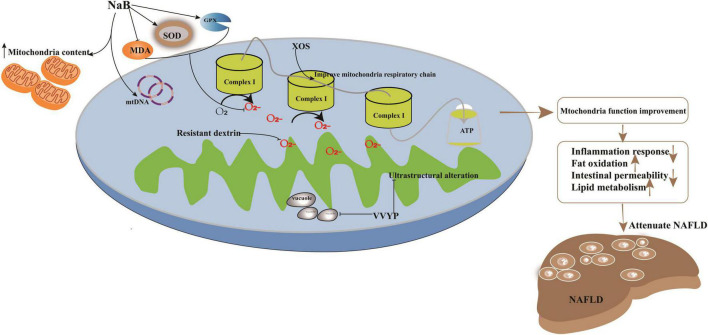
Potential targets in improvement of mitochondria function induced by imbalanced gut microbiota to attenuate NAFLD. Treatment strategy target to mitochondria had been explored. For example, application with sodium butyrate (NaB) can significantly enhance mitochondrial DNA content in HepG2 cell and increase the membrane potential function and the content of mitochondria to ameliorate its impaired dysfunction. NaB can elevate the activity of SOD and GPX and deplete the levels of prooxidative NOX2, ROS, and MDA; XOS enhanced the level of complex I and promote oxidative phosphorylation to improve mitochondrial respiratory capacity and evaluate the production of ATP; VVYP can decrease fragmentation of vacuolate and swollen mitochondria and improve the lipid metabolism.

Non-alcoholic fatty liver disease is characterized as a liver metabolic syndrome (MS), which is characterized by obesity, high blood levels of TG levels, low blood levels of high-density lipoprotein (HDL) cholesterol, and fasting glucose (72, 73). The HFD or HCFD dietary lifestyle obviously contributes to the alteration of these biochemical parameters to promote liver damage (73). However, a healthy metabolic status is important for cellular mitochondrial function (74). Mitochondria are the energy pool for continuous synthesis of adenosine triphosphate (ATP). Moreover, cellular ROS are mainly produced by the mitochondria. Subsequently, alteration of mitochondrial function can promote liver fat deposition, lipid peroxidation, hepatic oxidative stress, and liver insulin resistance (IR) (74). Recently, alteration of gut microbiota and its metabolites has been shown to induce accumulation of ROS in mitochondria and lead to alterations in oxidative stress and mitochondrial damage, which have been described in HS or NASH progression to fibrosis (75–77). In addition to metabolites such as SCFAs and BA, bacteria can also promote cross-talk between the microbiota and mitochondria by directly regulating the expression of cellular genes (Figure 2).

Previous investigations have illustrated members of *Bacteroides*, *Firmicutes*, and other bacteria alternating the level of SCFAs (78), which are utilized by the mitochondria to synthesize energy (79). For instance, the application of sodium butyrate (NaB) can significantly enhance mitochondrial DNA content in HepG2 cells, increase membrane potential function, and ameliorate mitochondrial dysfunction. Parallelly, NaB can elevate the activity of superoxide dismutase (SOD) and glutathione peroxidase (GPX) and deplete the levels of prooxidative NADPH oxidase 2 (NOX2), ROS, and malondialdehyde (MDA). Furthermore, deacetylation of histones may also be regulated by NaB to improve energy metabolism in NAFLD (80, 81).

In addition to SCFAs, BAs can influence mitochondrial energy metabolism and biogenesis. Bifidobacterium and Bacteroides are the main gut microbiota that transform conjugated BA into secondary BA during the progression of NAFLD in HFHC-fed or HFD-fed rats (38, 59). Secondary BAs regulate mitochondrial function by controlling transcription factors, including those involved in carbohydrate and lipid metabolism. For example, HFD-induced impairment of enterohepatic BA recycling was observed in the small intestine of HFD-fed rats (56). Consequently, the small heterodimer partner (SHP) and farnesoid X receptor (FXR) are robustly alternated (38). FXR is a target of sirtuin-1 (SIRT1), an NAD-dependent protein deacetylase, that regulates the expression of FXR (82) to regulate carbohydrate response element binding protein (ChREBP), steroid response element binding protein-1c (SREBP-1c), and PPAR-α to stimulate FA uptake and oxidation (83).

Supplementation with TMAVA promotes the development of HFD-induced NAFLD by influencing metabolic processes (4, 43). TMAVA decreases carnitine levels, which can be converted into acylcarnitine intermediates by carnitine acyltransferases involved in β-oxidation and the maintenance of mitochondrial structure. In contrast, TMAVA can inhibit endogenous carnitine synthesis and absorption in conjunction with γ-BB for BBOX binding. In addition, an HFD-fed diet altered the abundance of *Streptococcus*, *Globicatella*, *Leuconostoc*, *Lactococcus*, *Bifidobacterium*, and *Lactobacillus* in the report by Zheng et al. (84), which positively correlated with tryptophan, L-tyrosine, and skatole. Commonly, the gut microbiota metabolizes tryptophan into indoles, which promotes inflammation and causes severe HS. Therefore, the metabolites of the microbiota that benefit mitochondrial homeostasis should be further investigated to explore treatment strategies in patients with NAFLD.

## Microbiota and mitochondria: Potential therapeutic strategies in non-alcoholic fatty liver disease

Approaching NAFLD treatment on a molecular basis involving the microbiota and mitochondrial functions in tandem with lifestyle changes has proven promising. Traditionally, drugs cannot achieve adequate long-term effects and patients have presented with drug resistance. Exploring new therapeutic approaches, such as probiotics, prebiotics, symbiotics, or transplantation of fecal microbial communities, might overcome this limitation. We have identified some novel treatments targeting the microbiota and mitochondria for NAFLD, as shown in Table 3.

**TABLE 3:** Trials on the exploring of therapeutic strategy target to gut microbiota.

Study	Participant details and Treatment strategy	Effect of treatment
Zhao et al. (43)	Male C57BL/6J mice were fed an HFD for 8 weeks along with the indicated interventions. BBOX-knockout mice (BBOX^–/–^) were administered TMAVA.	Improve energy production and conversion, and carbohydrate transport and metabolism, and a decrease in lipid transport and metabolism. Improve hepatic mitochondria function
Xie et al. (12)	C57BL/6 mice were divided into four groups: CTL group, model (M) group, model + VVYP 10 mg/kg (M-VVYP), VVYP 10 mg/kg (VVYP).	VVYP inhibited the increased level of LPS and reversed the liver mitochondria dysfunction. VVYP increased the abundance of Eubacteriaceae, coriobacteriaceae, Desulfovibrionaceae, S24-7 and Bacteroidia VVYP reduced the abundance of Lactobacillus. VVYP conferred the protective effect of intestinal barrier via promoting the expression of the mucins and tight junction (TJ)-associated genes, inhibited subsequent liver inflammatory responses.
Craven et al. (1)	21 patients with NAFLD were randomly assigned to receive an allogenic or autologous FMT for 6 months post-transplant.	Allogenic FMT patients with elevated small intestinal permeability at baseline had a significant reduction 6 weeks after allogenic FMT. No significant changes in HOMA-IR or hepatic PDFF in patients who received the allogenic or autologous FMT.
Geng et al. (95)	Zebrafish model, and NAFLD rats model were divided into: control, high-fat (HF) diet, and HF diet plus different probiotics including ZW3.	Supplementation with ZW3 could improve the liver impairments and reduce inflammation through TLR4-MyD88 and JNK signaling pathways. ZW3 modulated gut microbiota by promoting relative abundance of Firmicutes and Lactobacillus, decreasing the abundance of Escherichia Shigella and Bacteroides.
Lensu et al. (49)	Male Wistar rats were divided into four dietary treatment groups: (1) HFD, (2) HFD supplemented with XOS, (3) control, and (4) LFD supplemented with XOS	XOS increased the growth of *F. prausnitzii*, lowered the level of cecal tyrosine, XOS decreased triglycerides on the. HFD, XOS increased the hepatic activity of β-HAD on the HFD, XOS supplementation seemed to ameliorate mitochondrial respiration injury induced by HFD, XOS had increased respiratory capacity available for the production of ATP through the electron flow from complex I, improved coupling of electron transport through complex I and oxidative phosphorylation. XOS diminished the epithelial injury caused by the HFD
Carbajo-Pescador et al. (56)	Wistar rats were separated into two subgroups (*n* = 24): Control, semi-purified high fat diet (HFD)	Exercise performance counteracted the HFD induced microbial imbalance, modifying *Firmicutes*/*Bacteroidetes* ratio.
Zhang et al. (84)	C57BL/6J male mice were divided into: normal diet, HFD, HFID (high-fat plus resistant dextrin diet)	Resistant dextrin ameliorated mitochondrial function and hepatic steatosis by manipulating the intestinal microbiota and its metabolites. Resistant dextrin supplementation via an HFID diet restored the structure of the intestinal microbiota, ameliorated microbial metabolic changes including to tryptophan and bile acid metabolism, decreased intestinal permeability and inflammatory cytokine levels, retained a healthy gut microenvironment, improved mitochondrial function, and ameliorated hepatic steatosis.
Li et al. (28)	Male C57BL/6 mice were divided into: normal diet, HFHC diet, HFHC diet supplemented with UDCA.	UDCA could ameliorate hepatic inflammation, and partially restore the dysbiosis of the gut microbiota for the treatment of NASH. UDCA could protect against intestinal barrier disruption and reduce serum levels of LPS and inflammatory cytokines in NASH mice.
Derosa et al. (89)	NAFLD patients were randomized to take placebo or VSL#3, 2 sachets/day for 3 months.	Tg, Hs-CRP, γ-GT, AST/ALT decrease, hepatic steatosis index (HSI) were improved.
Nor et al. (87)	NAFLD patients were supplemented with either a probiotics sachet (MCP^®^, BCMC^®^ strains) for 6 months.	Decrease the expression of CD8+ T lymphocytes and ZO-1 (Z-score), no improvement in the hepatic steatosis, fibrosis, and inflammatory activity scores.
Crommen et al. (86)	Obese patients with NAFLD received a combination of probiotic and a specific micronutrient mixture or a basic care micronutrient mixture for 12 weeks.	Improve serum AST, NAFLD fibrosis score, Tg and the visceral adiposity index.
Chong et al. (88)	Patients with NAFLD were randomly to take 2 sachets VSL#3^®^ probiotic or placebo twice daily for 10 weeks.	VSL#3^®^ probiotic supplementation did not significantly improve insulin resistance, endothelial dysfunction, oxidative stress, inflammation or liver injury in patients with NAFLD.

TMAVA, N,N,N-trimethyl-5-aminovaleric acid; HFD, high-fat diet; NC, normal chow; HFLC, high-fat/low-cholesterol diet; HFHC, high-fat/high-cholesterol diet; FMT, fecal microbiota transplantation; XOS, xylo-oligosaccharides; PDFF, hepatic proton density fat fraction; IR, insulin resistance; UDCA, ursodeoxycholic acid; TG, triglycerides; Hs-CRP, high-sensitivity C-reactive protein; γ-GT, transaminases and gamma-glutamyltransferase; HIS, hepatic steatosis index; HOMA-IR, insulin resistance.

### Probiotics

Probiotics are live organisms that benefit host health. They can stabilize the intestinal barrier, regulate immunomodulation, alter small intestinal bacterial overgrowth to inhibit inflammation in NAFLD, and contribute clinical benefits (85). Previous investigations have suggested that probiotics enhance the barrier function of epithelial cells and decrease intestinal permeability and endotoxemia in patients with liver disease. Probiotic therapy can significantly decrease the levels of ALT, AST, TC, HDL, and TNF-α and improve IR in patients with NASH (86). In addition, a randomized controlled study revealed that patients with NAFLD treated daily with *Lactobacillus* and *Bifidobacterium* species showed decreased levels of ALT and γGT, decreased counts of CD4+ T-cells and CD8+ T-cells, and improved HS fibrosis (87). Chong et al. (88) and Derosa et al. (89) found that NAFLD patients treated with VSL#3^®^ had a decreased AST/ALT ratio and hs-CRP level and improved IR and endothelial dysfunction. In addition, patients with NAFLD supplemented with metformin and probiotics showed improved serum AST and ALT levels and ultrasound grading of NASH (90). Furthermore, no studies have reported adverse effects during the study period, which suggests that this is a non-harmful therapeutic option for managing patients with NAFLD/NASH.

However, probiotic treatment could not improve all the alterations in biochemical indices induced by NAFLD; it could only attenuate some of them. For example, Chong et al. found that probiotic treatment did not improve the high levels of HDL, LDL, and TC in patients with NAFLD (88). Therefore, the clinical efficacy of probiotic therapies for NAFLD still needs to be confirmed in large-scale, multicenter clinical trials.

### Prebiotics

Xylo-oligosaccharides (XOS), a potential prebiotic target of *F. prausnitzii*, have been explored for the treatment of NAFLD induced by HFD diet (49). Lensu et al. (49) showed that XOS could increase the abundance of *F. prausnitzii*, which controlled hepatic fat content in humans (91). Moreover, XOS reduced intestinal inflammation by reversing the effects of HFD (92), enhanced hepatic β-HAD activity and complex I levels, and promoted oxidative phosphorylation, which improved mitochondrial respiratory capacity and elevated ATP production. In addition, XOS contributed to the improvement of hepatic fat oxidation by depleting the levels of tyrosine and isovalerate in NASH patients (93). ZW3, another probiotic strain isolated from Tibetan Kefir grains, a traditional Chinese food, improved the gut microbiota imbalance (94) and cleared the endotoxins produced by the gut microbiota and inflammasomes. It aided the resistance to infections induced by the methionine- and choline-deficient (MCD) diet by increasing the abundance of *Firmicutes*, *Lactobacillus*, and *Ruminococcaceae* and depleting that of *Escherichia*, *Shigella*, and *Bacteroides* (95). Moreover, ZW3 could repair the impaired intestinal mucosal barrier to significantly clear blood LPS in rats with NAFLD (96). In addition, supplementation with ZW3 also affected the levels of IL-4 and IL-10, which in turn attenuated the inflammatory response (95). Resistant dextrin (indigestible dextrin) is also a prebiotic explored for the treatment of NAFLD (84). Resistant dextrin ameliorated impaired mitochondrial function and cleared ROS by regulating the gut microbiota and improving HS in HFD-fed mice. Resistant dextrin helped maintain host liver health by increasing the abundance of *Blautia* and *Dubosiella*, which positively modulated the metabolite levels of dimethyl fumarate, lactitol, cafestol, and 4-hydroxyphenylacetic acid (97, 98). Resistant dextrin also prevented ROS accumulation, depleted secondary BAs and tryptophan, and enhanced the abundance of indole derivatives, which act as endogenous metabolites to reduce intestinal permeability and inflammatory responses in NAFLD (99–101). Active peptides or metabolites of the gut microbiota can also be used to improve the imbalance of gut microbiota induced by HFD (12, 28). For example, Val-Val-Tyr-Pro (VVYP) is a component of the globin digest (GD) (12), which can resist steatohepatitis by improving gut microbiota imbalance. For example, it can enhance the abundance of *Eubacteriaceae*, *Coriobacteriaceae*, *Desulfovibrionaceae*, and *Bacteroidia* to influence the levels of BAs and SCFAs. VVYP can also improve acetaldehyde-induced intestinal permeability and deplete endotoxemia to attenuate NAFLD. Interestingly, it is responsible for decreasing the fragmentation of vacuolate and swollen mitochondria and improving lipid metabolism by regulating LPS, TNF-α, and IL-6 (12).

### Fecal microbiota transplantation

Given the relationship between gut microbiota and NAFLD, another novel treatment approach–fecal microbiota transplantation (FMT)–has been explored to treat NAFLD (1). FMT from healthy donors increased the lactulose/mannitol ratio in 6 weeks in patients with NAFLD (102). Arguably, IR and the fatty liver phenotype improved by FMT in a mouse model (28). This result was also verified in 18 patients with MSs who were administered FMT (103). FMT can markedly alleviate lipid accumulation caused by estrogen deficiency within the liver tissue and attenuate the levels of ALT, AST, and TG in NAFLD. Finally, lifestyle changes can improve various chronic diseases (50). For example, exercise intervention effectively attenuated hepatic homeostasis damage and imbalanced gut microbiota induced by HFD. Exercise can repair the intestinal barrier disrupted by HFD, suppress oxidative stress, gut–liver axis activation, and inflammation, and modulate BA metabolism and enterohepatic circulation to attenuate the progression of NAFLD (56).

### Lifestyle intervention

Although many studies have explored the efficacy of probiotics, prebiotics, or FMT in the treatment of NAFLD, physical activity and dietary modifications are the only effective therapeutic options for NAFLD management, which have been confirmed to be associated with the modulation of gut microbiota and its metabolites (9). For instance, participants with obesity and NAFLD, who were administered an isocaloric low-carbohydrate diet as the intervention, showed improved fatty liver metabolism and rapid shifts in the composition of the gut microbiota. This was achieved via decreasing hepatic *de novo* lipogenesis, increasing serum β-hydroxybutyrate concentrations and mitochondrial β-oxidation, and rapidly increasing Streptococcus abundance (104). Regular aerobic exercise intervention also increased gut microbial diversity and altered the composition and functional capacity of the gut microbiota in participants with NAFLD (105). Calabrese et al. investigated the impact of different lifestyle interventions on the composition of the gut microbiota in NAFLD, including a low glycemic index Mediterranean diet (LGIMD), aerobic activity program (ATFIS_1), and LGIMD plus ATFIS_1. Consequently, different microbiota alterations were observed according to the different lifestyles. For example, the abundance of *Ruminococcus*, *Oscillospiraceae*-UCG002, *Oscillospiraceae*-UCG005, *Dialister*, *Alistipes*, and *Eubacterium* eligens showed an increasing trend, whereas that of *Collinsella* showed a decreasing trend in LGIMD-ATFIS_1 intervention, which was not found in other groups (106). In addition, a randomized controlled trial revealed that microbial diversity deteriorates with increased hepatic fat content, while exercise and diet may help maintain the diversity of the gut microbiota (107). The abundance of *Ruminococcus*, *Bacteroides*, and *Lachnospiraceae* (ASV5361) increased during both exercise and dietary interventions. However, some ASVs isolated from the same family or genus exhibit different behaviors. For example, the abundance of ASV 4432, a *Lachnospiraceae*, increased by aerobic exercise combined with diet but decreased after fiber-enriched low-carbohydrate diet intervention. Therefore, lifestyle therapies based on dietary or exercise interventions should be personalized according to ethnicity, eating habits, and exercise habits.

## Limitations and future prospects

The prevalence of NAFLD has become an alarming pandemic, contributing to a high social burden as well as an increased risk of morbidity and mortality. Previous studies have comprehensively hinted at the influence of gut microbiota on the pathogenesis of NAFLD through their involvement in metabolic pathway alteration, inflammatory damage, or immune response. Simultaneously, the metabolites generated by the microbiota, including SCFAs, BAs, and TML, also participate in the modulation of liver health homeostasis by cross-talk with mitochondria. Emerging evidence suggests that novel treatment strategy targets for NAFLD may focus on gut microbiota–mitochondria cross-talk to modulate liver health homeostasis. Recent studies have investigated novel therapeutic interventions, including prebiotics, probiotics, and metabolites, to modulate imbalances in the gut microbiota, improve mitochondrial biogenesis function, and inhibit the accumulation of ROS.

However, there are several limitations in NAFLD therapy that target the microbiota. The baseline gut microbiota is generally influenced by numerous factors, including age, sex, race, geographic location, diet, and lifestyle (108). Moreover, the gut microbiome of an individual is dynamic. Consequently, establishing causality in microbiome-NAFLD host interactions remains challenging. More effort is needed to explore potential microbiota markers related to NAFLD. Another challenge is that not all patients respond to intervention or treatment in a similar manner during the construction of the relationship between the gut microbiota and the improvement of NAFLD. Previous reports have revealed that individual variations exist in response to exercise intervention (107). As a result, some patients with low or no response may show more improvement than others. Therefore, the establishment of an effective intervention strategy should be based on the evaluation of individual microbiota, as well as the differences between responders and low/non-responders to various interventions. Furthermore, the need to predict the responsiveness of each subject to develop personalized pre- or probiotic treatment, dietary, or lifestyle intervention is of great clinical importance. Currently, the application of these novel treatments for NAFLD is still in its early stages. More large-scale multicenter studies on microbiota–mitochondria cross-talk are required to confirm and illustrate the mechanisms and potential treatment effects in NAFLD.

## Author contributions

QZ and WG were responsible for the design and writing of this work. QW and ZT performed the literature search. YW was responsible for the figure design. WX contributed to the revised manuscript. All authors reviewed this draft and approved the final manuscript.

## References

[1] Craven L Rahman A Nair Parvathy S Beaton M Silverman J Qumosani K Allogenic fecal microbiota transplantation in patients with nonalcoholic fatty liver disease improves abnormal small intestinal permeability: a randomized control trial. *Am J Gastroenterol.* (2020) 115:1055–65. 10.14309/ajg.0000000000000661 32618656

[2] Nawrot M Peschard S Lestavel S Staels B. Intestine-liver crosstalk in type 2 diabetes and non-alcoholic fatty liver disease. *Metabolism.* (2021) 123:154844. 10.1016/j.metabol.2021.154844 34343577

[3] Ponziani FR Bhoori S Castelli C Putignani L Rivoltini L Del Chierico F Hepatocellular carcinoma is associated with gut microbiota profile and inflammation in nonalcoholic fatty liver disease. *Hepatology.* (2019) 69:107–20. 29665135 10.1002/hep.30036

[4] Zeybel M Arif M Li X Altay O Yang H Shi M Multiomics analysis reveals the impact of microbiota on host metabolism in hepatic steatosis. *Adv Sci (Weinh).* (2022) 9:e2104373. 10.1002/advs.202104373 35128832 PMC9008426

[5] Aron-Wisnewsky J Warmbrunn MV Nieuwdorp M Clement K. Nonalcoholic fatty liver disease: modulating gut microbiota to improve severity? *Gastroenterology.* (2020) 158:1881–98. 32044317 10.1053/j.gastro.2020.01.049

[6] Bauer KC Littlejohn PT Ayala V Creus-Cuadros A Finlay BB. Nonalcoholic fatty liver disease and the gut-liver axis: exploring an undernutrition perspective. *Gastroenterology.* (2022) 162:1858.e–75.e. 10.1053/j.gastro.2022.01.058 35248539

[7] Turnbaugh PJ Ley RE Hamady M Fraser-Liggett CM Knight R Gordon JI. The human microbiome project. *Nature.* (2007) 449:804–10. 17943116 10.1038/nature06244 PMC3709439

[8] Sbierski-Kind J Grenkowitz S Schlickeiser S Sandforth A Friedrich M Kunkel D Effects of caloric restriction on the gut microbiome are linked with immune senescence. *Microbiome.* (2022) 10:57. 35379337 10.1186/s40168-022-01249-4 PMC8978410

[9] Azzoni R Marsland BJ. The lung-brain axis: a new frontier in host-microbe interactions. *Immunity.* (2022) 55:589–91. 10.1016/j.immuni.2022.03.015 35417673

[10] Feng P Li Q Liu L Wang S Wu Z Tao Y Crocetin prolongs recovery period of DSS-induced colitis via altering intestinal microbiome and increasing intestinal permeability. *Int J Mol Sci.* (2022) 23:3832. 10.3390/ijms23073832 35409192 PMC8998954

[11] Lang S Schnabl B. Microbiota and fatty liver disease-the known, the unknown, and the future. *Cell Host Microbe.* (2020) 28:233–44. 10.1016/j.chom.2020.07.007 32791115 PMC7467841

[12] Xie X Zhang L Yuan S Li H Zheng C Xie S Val-Val-Tyr-Pro protects against non-alcoholic steatohepatitis in mice by modulating the gut microbiota and gut-liver axis activation. *J Cell Mol Med.* (2021) 25:1439–55. 10.1111/jcmm.16229 33400402 PMC7875918

[13] Wang Z Zeng M Wang Z Qin F Chen J He Z. Dietary polyphenols to combat nonalcoholic fatty liver disease via the gut-brain-liver axis: a review of possible mechanisms. *J Agric Food Chem.* (2021) 69:3585–600. 10.1021/acs.jafc.1c00751 33729777

[14] Gesper M Nonnast ABH Kumowski N Stoehr R Schuett K Marx N Gut-derived metabolite indole-3-propionic acid modulates mitochondrial function in cardiomyocytes and alters cardiac function. *Front Med (Lausanne).* (2021) 8:648259. 10.3389/fmed.2021.648259 33829028 PMC8019752

[15] Singh R Zogg H Wei L Bartlett A Ghoshal UC Rajender S Gut microbial dysbiosis in the pathogenesis of gastrointestinal dysmotility and metabolic disorders. *J Neurogastroenterol Motil.* (2021) 27:19–34. 10.5056/jnm20149 33166939 PMC7786094

[16] Li H Yuan W Tian Y Tian F Wang Y Sun X Eugenol alleviated nonalcoholic fatty liver disease in rat via a gut-brain-liver axis involving glucagon-like Peptide-1. *Arch Biochem Biophys.* (2022) 725:109269. 10.1016/j.abb.2022.109269 35508252

[17] Beraza N Trautwein C. The gut-brain-liver axis: a new option to treat obesity and diabetes? *Hepatology.* (2008) 48:1011–3. 10.1002/hep.22478 18752321

[18] Huang Y Xin W Xiong J Yao M Zhang B Zhao J. The intestinal microbiota and metabolites in the gut-kidney-heart axis of chronic kidney disease. *Front Pharmacol.* (2022) 13:837500. 35370631 10.3389/fphar.2022.837500 PMC8971625

[19] Olubodun-Obadun TG Ishola IO Adeyemi OO. Impact of environmental toxicants exposure on gut-brain axis in Parkinson disease. *Drug Metab Pers Ther.* (2022). [Epub ahead of print]. 10.1515/dmpt-2021-0144 35377569

[20] Peng H Yu S Zhang Y Yin Y Zhou J. Intestinal dopamine receptor D2 is required for neuroprotection against 1-methyl-4-phenyl-1,2,3,6-tetrahydropyridine-induced dopaminergic neurodegeneration. *Neurosci Bull.* (2022) 38:871–86. 10.1007/s12264-022-00848-3 35399136 PMC9352842

[21] Zhu Y Li Y Zhang Q Song Y Wang L Zhu Z. Interactions between intestinal microbiota and neural mitochondria: a new perspective on communicating pathway from gut to brain. *Front Microbiol.* (2022) 13:798917. 10.3389/fmicb.2022.798917 35283843 PMC8908256

[22] Du M Zheng M Liu A Wang L Pan X Liu J Effects of emerging contaminants and heavy metals on variation in bacterial communities in estuarine sediments. *Sci Total Environ.* (2022) 832:155118. 10.1016/j.scitotenv.2022.155118 35398136

[23] Vezza T Abad-Jiménez Z Marti-Cabrera M Rocha M Víctor VM. Microbiota-mitochondria inter-talk: a potential therapeutic strategy in obesity and type 2 diabetes. *Antioxidants (Basel).* (2020) 9:848. 10.3390/antiox9090848 32927712 PMC7554719

[24] Silveira MAD Bilodeau S Greten TF Wang XW Trinchieri G. The gut-liver axis: host microbiota interactions shape hepatocarcinogenesis. *Trends Cancer.* (2022) 8:583–97. 10.1016/j.trecan.2022.02.009 35331674 PMC9232941

[25] Helsley RN Miyata T Kadam A Varadharajan V Sangwan N Huang EC Gut microbial trimethylamine is elevated in alcohol-associated hepatitis and contributes to ethanol-induced liver injury in mice. *Elife.* (2022) 11:e76554. 10.7554/eLife.76554 35084335 PMC8853661

[26] Ding Q Guo R Pei L Lai S Li J Yin Y N-Acetylcysteine alleviates high fat diet-induced hepatic steatosis and liver injury via regulating the intestinal microecology in mice. *Food Funct.* (2022) 13:3368–80. 10.1039/d1fo03952k 35229847

[27] Magne F Gotteland M Gauthier L Zazueta A Pesoa S Navarrete P The firmicutes/bacteroidetes ratio: a relevant marker of gut dysbiosis in obese patients? *Nutrients.* (2020) 12:1474. 10.3390/nu12051474 32438689 PMC7285218

[28] Li H Wang Q Chen P Zhou C Zhang X Chen L. Ursodeoxycholic acid treatment restores gut microbiota and alleviates liver inflammation in non-alcoholic steatohepatitic mouse model. *Front Pharmacol.* (2021) 12:788558. 10.3389/fphar.2021.788558 34938193 PMC8685972

[29] Caussy C Tripathi A Humphrey G Bassirian S Singh S Faulkner C A gut microbiome signature for cirrhosis due to nonalcoholic fatty liver disease. *Nat Commun.* (2019) 10:1406. 30926798 10.1038/s41467-019-09455-9 PMC6440960

[30] Song M Yuan F Li X Ma X Yin X Rouchka EC Analysis of sex differences in dietary copper-fructose interaction-induced alterations of gut microbial activity in relation to hepatic steatosis. *Biol Sex Differ.* (2021) 12:3. 10.1186/s13293-020-00346-z 33407877 PMC7789350

[31] Pafco B Sharma AK Petrzelkova KJ Vlckova K Todd A Yeoman CJ Gut microbiome composition of wild western lowland gorillas is associated with individual age and sex factors. *Am J Phys Anthropol.* (2019) 169:575–85. 10.1002/ajpa.23842 31025322

[32] Min Y Ma X Sankaran K Ru Y Chen L Baiocchi M Sex-specific association between gut microbiome and fat distribution. *Nat Commun.* (2019) 10:2408. 31160598 10.1038/s41467-019-10440-5 PMC6546740

[33] Shi J Yang Y Xu W Cai H Wu J Long J Sex-specific associations between gut microbiome and non-alcoholic fatty liver disease among urban chinese adults. *Microorganisms.* (2021) 9:2118. 10.3390/microorganisms9102118 34683439 PMC8537656

[34] Behari J Graham L Wang R Schirda C Borhani AA Methe BA Dynamics of hepatic steatosis resolution and changes in gut microbiome with weight loss in nonalcoholic fatty liver disease. *Obes Sci Pract.* (2021) 7:217–25. 10.1002/osp4.476 33841891 PMC8019274

[35] Santos-Marcos JA Haro C Vega-Rojas A Alcala-Diaz JF Molina-Abril H Leon-Acuna A Sex differences in the gut microbiota as potential determinants of gender predisposition to disease. *Mol Nutr Food Res.* (2019) 63:e1800870. 30636111 10.1002/mnfr.201800870

[36] Li P Yan K Chang X Chen X Wang R Fan X Sex-specific maternal calcium requirements for the prevention of nonalcoholic fatty liver disease by altering the intestinal microbiota and lipid metabolism in the high-fat-diet-fed offspring mice. *Gut Microbes.* (2020) 11:1590–607. 10.1080/19490976.2020.1768645 32576050 PMC7524148

[37] Brown ZJ Gregory S Hewitt DB Iacono S Choe J Labiner HE Safety, efficacy, and tolerability of immune checkpoint inhibitors in the treatment of hepatocellular carcinoma. *Surg Oncol.* (2022) 42:101748. 35395582 10.1016/j.suronc.2022.101748

[38] Zhang X Coker OO Chu ES Fu K Lau HCH Wang YX Dietary cholesterol drives fatty liver-associated liver cancer by modulating gut microbiota and metabolites. *Gut.* (2021) 70:761–74. 10.1136/gutjnl-2019-319664 32694178 PMC7948195

[39] Behary J Amorim N Jiang XT Raposo A Gong L McGovern E Gut microbiota impact on the peripheral immune response in non-alcoholic fatty liver disease related hepatocellular carcinoma. *Nat Commun.* (2021) 12:187. 10.1038/s41467-020-20422-7 33420074 PMC7794332

[40] Pettinelli P Arendt BM Schwenger KJP Sivaraj S Bhat M Comelli EM Relationship between hepatic gene expression, intestinal microbiota and inferred functional metagenomic analysis in NAFLD. *Clin Transl Gastroenterol.* (2022) 13:e00466. 10.14309/ctg.0000000000000466 35166723 PMC10476782

[41] Gallego O Ruiz FX Ardevol A Dominguez M Alvarez R de Lera AR Structural basis for the high all-trans-retinaldehyde reductase activity of the tumor marker AKR1B10. *Proc Natl Acad Sci USA.* (2007) 104:20764–9. 10.1073/pnas.0705659105 18087047 PMC2410076

[42] Baranova A Schlauch K Elariny H Jarrar M Bennett C Nugent C Gene expression patterns in hepatic tissue and visceral adipose tissue of patients with non-alcoholic fatty liver disease. *Obes Surg.* (2007) 17:1111–8. 17953248 10.1007/s11695-007-9187-y

[43] Zhao M Zhao L Xiong X He Y Huang W Liu Z TMAVA, a metabolite of intestinal microbes, is increased in plasma from patients with liver steatosis, inhibits γ-butyrobetaine hydroxylase, and exacerbates fatty liver in mice. *Gastroenterology.* (2020) 158:2266.e–81.e. 10.1053/j.gastro.2020.02.033 32105727

[44] Li XS Wang Z Cajka T Buffa JA Nemet I Hurd AG Untargeted metabolomics identifies trimethyllysine, a TMAO-producing nutrient precursor, as a predictor of incident cardiovascular disease risk. *JCI Insight.* (2018) 3:e99096. 10.1172/jci.insight.99096 29563342 PMC5926943

[45] Shekhawat PS Sonne S Carter AL Matern D Ganapathy V. Enzymes involved in L-carnitine biosynthesis are expressed by small intestinal enterocytes in mice: implications for gut health. *J Crohns Colitis.* (2013) 7:e197–205. 10.1016/j.crohns.2012.08.011 22999781 PMC3644392

[46] Cummings JH Pomare EW Branch WJ Naylor CP Macfarlane GT. Short chain fatty acids in human large intestine, portal, hepatic and venous blood. *Gut.* (1987) 28:1221–7. 3678950 10.1136/gut.28.10.1221 PMC1433442

[47] Boursier J Mueller O Barret M Machado M Fizanne L Araujo-Perez F The severity of nonalcoholic fatty liver disease is associated with gut dysbiosis and shift in the metabolic function of the gut microbiota. *Hepatology.* (2016) 63:764–75. 10.1002/hep.28356 26600078 PMC4975935

[48] Michail S Lin M Frey MR Fanter R Paliy O Hilbush B Altered gut microbial energy and metabolism in children with non-alcoholic fatty liver disease. *FEMS Microbiol Ecol.* (2015) 91:1–9. 10.1093/femsec/fiu002 PMC4358749 25764541

[49] Lensu S Pariyani R Mäkinen E Yang B Saleem W Munukka E Prebiotic Xylo-oligosaccharides ameliorate high-fat-diet-induced hepatic steatosis in rats. *Nutrients.* (2020) 12:3225. 10.3390/nu12113225 33105554 PMC7690286

[50] Liu L Fu Q Li T Shao K Zhu X Cong Y Gut microbiota and butyrate contribute to nonalcoholic fatty liver disease in premenopause due to estrogen deficiency. *PLoS One.* (2022) 17:e0262855. 10.1371/journal.pone.0262855 35108315 PMC8809533

[51] Ding Y Yanagi K Cheng C Alaniz RC Lee K Jayaraman A. Interactions between gut microbiota and non-alcoholic liver disease: the role of microbiota-derived metabolites. *Pharmacol Res.* (2019) 141:521–9. 30660825 10.1016/j.phrs.2019.01.029 PMC6392453

[52] Smith PM Howitt MR Panikov N Michaud M Gallini CA Bohlooly YM The microbial metabolites, short-chain fatty acids, regulate colonic Treg cell homeostasis. *Science.* (2013) 341:569–73. 23828891 10.1126/science.1241165 PMC3807819

[53] Sun M Wu W Chen L Yang W Huang X Ma C Microbiota-derived short-chain fatty acids promote Th1 cell IL-10 production to maintain intestinal homeostasis. *Nat Commun.* (2018) 9:3555. 10.1038/s41467-018-05901-2 30177845 PMC6120873

[54] Singh V Yeoh BS Chassaing B Xiao X Saha P Aguilera Olvera R Dysregulated microbial fermentation of soluble fiber induces cholestatic liver cancer. *Cell.* (2018) 175:679.e–94.e. 30340040 10.1016/j.cell.2018.09.004 PMC6232850

[55] Pant K Yadav AK Gupta P Islam R Saraya A Venugopal SK. Butyrate induces ROS-mediated apoptosis by modulating miR-22/SIRT-1 pathway in hepatic cancer cells. *Redox Biol.* (2017) 12:340–9. 10.1016/j.redox.2017.03.006 28288414 PMC5350572

[56] Carbajo-Pescador S Porras D Garcia-Mediavilla MV Martinez-Florez S Juarez-Fernandez M Cuevas MJ Beneficial effects of exercise on gut microbiota functionality and barrier integrity, and gut-liver crosstalk in an in vivo model of early obesity and non-alcoholic fatty liver disease. *Dis Model Mech.* (2019) 12:dmm039206. 10.1242/dmm.039206 30971408 PMC6550047

[57] Puri P Daita K Joyce A Mirshahi F Santhekadur PK Cazanave S The presence and severity of nonalcoholic steatohepatitis is associated with specific changes in circulating bile acids. *Hepatology.* (2018) 67:534–48. 28696585 10.1002/hep.29359 PMC5764808

[58] Zhu L Baker SS Gill C Liu W Alkhouri R Baker RD Characterization of gut microbiomes in nonalcoholic steatohepatitis (NASH) patients: a connection between endogenous alcohol and NASH. *Hepatology.* (2013) 57:601–9. 10.1002/hep.26093 23055155

[59] Jia W Xie G Jia W. Bile acid-microbiota crosstalk in gastrointestinal inflammation and carcinogenesis. *Nat Rev Gastroenterol Hepatol.* (2018) 15:111–28. 29018272 10.1038/nrgastro.2017.119 PMC5899973

[60] Vyas D Kadegowda AK Erdman RA. Dietary conjugated linoleic Acid and hepatic steatosis: species-specific effects on liver and adipose lipid metabolism and gene expression. *J Nutr Metab.* (2012) 2012:932928. 10.1155/2012/932928 21869929 PMC3160137

[61] Sun B Jia Y Hong J Sun Q Gao S Hu Y Sodium butyrate ameliorates high-fat-diet-induced non-alcoholic fatty liver disease through peroxisome proliferator-activated receptor α-mediated activation of β oxidation and suppression of inflammation. *J Agric Food Chem.* (2018) 66:7633–42. 10.1021/acs.jafc.8b01189 29961332

[62] Zhou D Chen YW Zhao ZH Yang RX Xin FZ Liu XL Sodium butyrate reduces high-fat diet-induced non-alcoholic steatohepatitis through upregulation of hepatic GLP-1R expression. *Exp Mol Med.* (2018) 50:1–12. 10.1038/s12276-018-0183-1 30510243 PMC6277380

[63] Caussy C Hsu C Lo MT Liu A Bettencourt R Ajmera VH Link between gut-microbiome derived metabolite and shared gene-effects with hepatic steatosis and fibrosis in NAFLD. *Hepatology.* (2018) 68:918–32. 10.1002/hep.29892 29572891 PMC6151296

[64] Beloborodova N Bairamov I Olenin A Shubina V Teplova V Fedotcheva N. Effect of phenolic acids of microbial origin on production of reactive oxygen species in mitochondria and neutrophils. *J Biomed Sci.* (2012) 19:89. 10.1186/1423-0127-19-89 23061754 PMC3503878

[65] Wang TY Tao SY Wu YX An T Lv BH Liu JX Quinoa reduces high-fat diet-induced obesity in mice via potential microbiota-gut-brain-liver interaction mechanisms. *Microbiol Spectr.* (2022) 10:e0032922. 10.1128/spectrum.00329-22 35583337 PMC9241864

[66] Trapecar M Wogram E Svoboda D Communal C Omer A Lungjangwa T Human physiomimetic model integrating microphysiological systems of the gut, liver, and brain for studies of neurodegenerative diseases. *Sci Adv.* (2021) 7:eabd1707. 10.1126/sciadv.abd1707 33514545 PMC7846169

[67] Milanski M Arruda AP Coope A Ignacio-Souza LM Nunez CE Roman EA Inhibition of hypothalamic inflammation reverses diet-induced insulin resistance in the liver. *Diabetes.* (2012) 61:1455–62. 10.2337/db11-0390 22522614 PMC3357298

[68] Ryan D Acosta A. GLP-1 receptor agonists: nonglycemic clinical effects in weight loss and beyond. *Obesity (Silver Spring).* (2015) 23:1119–29. 10.1002/oby.21107 25959380 PMC4692091

[69] de Faria Ghetti F Oliveira DG de Oliveira JM de Castro Ferreira L Cesar DE Moreira APB. Influence of gut microbiota on the development and progression of nonalcoholic steatohepatitis. *Eur J Nutr.* (2018) 57:861–76. 28875318 10.1007/s00394-017-1524-x

[70] Higarza SG Arboleya S Gueimonde M Gomez-Lazaro E Arias JL Arias N. Neurobehavioral dysfunction in non-alcoholic steatohepatitis is associated with hyperammonemia, gut dysbiosis, and metabolic and functional brain regional deficits. *PLoS One.* (2019) 14:e0223019. 10.1371/journal.pone.0223019 31539420 PMC6754158

[71] Cerreto M Santopaolo F Gasbarrini A Pompili M Ponziani FR. Bariatric surgery and liver disease: general considerations and role of the gut-liver axis. *Nutrients.* (2021) 13:2649. 10.3390/nu13082649 34444807 PMC8399840

[72] Yasutake K Kohjima M Kotoh K Nakashima M Nakamuta M Enjoji M. Dietary habits and behaviors associated with nonalcoholic fatty liver disease. *World J Gastroenterol.* (2014) 20:1756–67. 24587653 10.3748/wjg.v20.i7.1756 PMC3930974

[73] Ziolkowska S Binienda A Jabłkowski M Szemraj J Czarny P. The interplay between insulin resistance, inflammation, oxidative stress, base excision repair and metabolic syndrome in nonalcoholic fatty liver disease. *Int J Mol Sci.* (2021) 22:11128. 10.3390/ijms222011128 34681787 PMC8537238

[74] La Colla A Cámara CA Campisano S Chisari AN. Mitochondrial dysfunction and epigenetics underlying the link between early-life nutrition and non-alcoholic fatty liver disease. *Nutr Res Rev.* (2022):1–33. [Epub ahead of print]. 10.1017/S0954422422000038 35067233

[75] Borrelli A Bonelli P Tuccillo FM Goldfine ID Evans JL Buonaguro FM Role of gut microbiota and oxidative stress in the progression of non-alcoholic fatty liver disease to hepatocarcinoma: current and innovative therapeutic approaches. *Redox Biol.* (2018) 15:467–79. 10.1016/j.redox.2018.01.009 29413959 PMC5975181

[76] Peverill W Powell LW Skoien R. Evolving concepts in the pathogenesis of NASH: beyond steatosis and inflammation. *Int J Mol Sci.* (2014) 15:8591–638. 10.3390/ijms15058591 24830559 PMC4057750

[77] Tilg H Moschen AR. Evolution of inflammation in nonalcoholic fatty liver disease: the multiple parallel hits hypothesis. *Hepatology.* (2010) 52:1836–46. 10.1002/hep.24001 21038418

[78] Yan J Xue Q Chen W Wang K Peng D Jiang J Probiotic-fermented rice buckwheat alleviates high-fat diet-induced hyperlipidemia in mice by suppressing lipid accumulation and modulating gut microbiota. *Food Res Int.* (2022) 155:111125. 10.1016/j.foodres.2022.111125 35400410

[79] Lumeng L Davis EJ. The oxidation of acetate by liver mitochondria. *FEBS Lett.* (1973) 29:124–6. 4352503 10.1016/0014-5793(73)80541-1

[80] Zhao T Gu J Zhang H Wang Z Zhang W Zhao Y Sodium butyrate-modulated mitochondrial function in high-insulin induced HepG2 cell dysfunction. *Oxid Med Cell Longev.* (2020) 2020:1904609. 10.1155/2020/1904609 32724489 PMC7382753

[81] Mihaylova MM Vasquez DS Ravnskjaer K Denechaud PD Yu RT Alvarez JG Class IIa histone deacetylases are hormone-activated regulators of FOXO and mammalian glucose homeostasis. *Cell.* (2011) 145:607–21. 10.1016/j.cell.2011.03.043 21565617 PMC3117637

[82] Kuipers F Bloks VW Groen AK. Beyond intestinal soap–bile acids in metabolic control. *Nat Rev Endocrinol.* (2014) 10:488–98. 10.1038/nrendo.2014.60 24821328

[83] Joyce SA Gahan CG. Bile acid modifications at the microbe-host interface: potential for nutraceutical and pharmaceutical interventions in host health. *Annu Rev Food Sci Technol.* (2016) 7:313–33. 10.1146/annurev-food-041715-033159 26772409

[84] Zhang Z Chen X Cui B. Modulation of the fecal microbiome and metabolome by resistant dextrin ameliorates hepatic steatosis and mitochondrial abnormalities in mice. *Food Funct.* (2021) 12:4504–18. 10.1039/d1fo00249j 33885128

[85] Rodrigo T Dulani S Nimali Seneviratne S De Silva AP Fernando J De Silva HJ Effects of probiotics combined with dietary and lifestyle modification on clinical, biochemical, and radiological parameters in obese children with nonalcoholic fatty liver disease/nonalcoholic steatohepatitis: a randomized clinical trial. *Clin Exp Pediatr.* (2022) 65:304–11. 10.3345/cep.2021.00787 34773939 PMC9171460

[86] Crommen S Rheinwalt KP Plamper A Simon MC Rosler D Fimmers R Specifically tailored multistrain probiotic and micronutrient mixture affects nonalcoholic fatty liver disease-related markers in patients with obesity after mini gastric bypass surgery. *J Nutr.* (2022) 152:408–18. 10.1093/jn/nxab392 34919684

[87] Nor FHM Abdullah S Ibrahim Z Nor MHM Osman MI Al Farraj DA Role of extremophilic Bacillus cereus KH1 and its lipopeptide in treatment of organic pollutant in wastewater. *Bioprocess Biosyst Eng.* (2022). [Epub ahead of print]. 10.1007/s00449-022-02749-1 35779113

[88] Chong PL Laight D Aspinall RJ Higginson A Cummings MH. A randomised placebo controlled trial of VSL#3((R)) probiotic on biomarkers of cardiovascular risk and liver injury in non-alcoholic fatty liver disease. *BMC Gastroenterol.* (2021) 21:144. 10.1186/s12876-021-01660-5 33794784 PMC8015038

[89] Derosa G Guasti L D’Angelo A Martinotti C Valentino MC Di Matteo S Probiotic therapy with VSL#3((R)) in patients with NAFLD: a randomized clinical trial. *Front Nutr.* (2022) 9:846873. 10.3389/fnut.2022.846873 35685888 PMC9172906

[90] Aller R De Luis DA Izaola O Conde R Gonzalez Sagrado M Primo D Effect of a probiotic on liver aminotransferases in nonalcoholic fatty liver disease patients: a double blind randomized clinical trial. *Eur Rev Med Pharmacol Sci.* (2011) 15:1090–5. 22013734

[91] Munukka E Pekkala S Wiklund P Rasool O Borra R Kong L Gut-adipose tissue axis in hepatic fat accumulation in humans. *J Hepatol.* (2014) 61:132–8. 10.1016/j.jhep.2014.02.020 24613361

[92] Gulhane M Murray L Lourie R Tong H Sheng YH Wang R High fat diets induce colonic epithelial cell stress and inflammation that is reversed by IL-22. *Sci Rep.* (2016) 6:28990. 10.1038/srep28990 27350069 PMC4924095

[93] Gitto S Schepis F Andreone P Villa E. Study of the serum metabolomic profile in nonalcoholic fatty liver disease: research and clinical perspectives. *Metabolites.* (2018) 8:17. 10.3390/metabo8010017 PMC5876006 29495258

[94] Xing Z Tang W Yang Y Geng W Rehman RU Wang Y. Colonization and gut flora modulation of *Lactobacillus kefiranofaciens* ZW3 in the intestinal tract of mice. *Probiotics Antimicrob Proteins.* (2018) 10:374–82. 10.1007/s12602-017-9288-4 28578494

[95] Geng W Zhang Y Yang J Zhang J Zhao J Wang J Identification of a novel probiotic and its protective effects on NAFLD via modulating gut microbial community. *J Sci Food Agric.* (2022) 102:4620–8. 10.1002/jsfa.11820 35174500

[96] Gorina R Font-Nieves M Márquez-Kisinousky L Santalucia T Planas AM. Astrocyte TLR4 activation induces a proinflammatory environment through the interplay between MyD88-dependent NFκB signaling, MAPK, and Jak1/Stat1 pathways. *Glia.* (2011) 59:242–55. 10.1002/glia.21094 21125645

[97] Dwivedi DK Jena G Kumar V. Dimethyl fumarate protects thioacetamide-induced liver damage in rats: studies on Nrf2, NLRP3, and NF-κB. *J Biochem Mol Toxicol.* (2020) 34:e22476. 10.1002/jbt.22476 32060995

[98] Zhao H Jiang Z Chang X Xue H Yahefu W Zhang X. 4-Hydroxyphenylacetic acid prevents acute APAP-induced liver injury by increasing phase II and antioxidant enzymes in mice. *Front Pharmacol.* (2018) 9:653. 10.3389/fphar.2018.00653 29973881 PMC6020787

[99] Wlodarska M Luo C Kolde R d’Hennezel E Annand JW Heim CE Indoleacrylic acid produced by commensal *Peptostreptococcus* species suppresses inflammation. *Cell Host Microbe.* (2017) 22:25–37.e. 10.1016/j.chom.2017.06.007 28704649 PMC5672633

[100] Lee Y Kwon EY Choi MS. Dietary isoliquiritigenin at a low dose ameliorates insulin resistance and NAFLD in diet-induced obesity in C57BL/6J mice. *Int J Mol Sci.* (2018) 19:3281. 10.3390/ijms19103281 30360437 PMC6214092

[101] Park SM Lee JR Ku SK Cho IJ Byun SH Kim SC Isoliquiritigenin in licorice functions as a hepatic protectant by induction of antioxidant genes through extracellular signal-regulated kinase-mediated NF-E2-related factor-2 signaling pathway. *Eur J Nutr.* (2016) 55:2431–44. 10.1007/s00394-015-1051-6 26593436

[102] Kushwaha V Rai P Varshney S Gupta S Khandelwal N Kumar D Sodium butyrate reduces endoplasmic reticulum stress by modulating CHOP and empowers favorable anti-inflammatory adipose tissue immune-metabolism in HFD fed mice model of obesity. *Food Chem (Oxf).* (2022) 4:100079. 10.1016/j.fochms.2022.100079 35415672 PMC8991629

[103] Vrieze A Van Nood E Holleman F Salojärvi J Kootte RS Bartelsman JF Transfer of intestinal microbiota from lean donors increases insulin sensitivity in individuals with metabolic syndrome. *Gastroenterology.* (2012) 143:913.e–6.e. 22728514 10.1053/j.gastro.2012.06.031

[104] Mardinoglu A Wu H Bjornson E Zhang C Hakkarainen A Rasanen SM An integrated understanding of the rapid metabolic benefits of a carbohydrate-restricted diet on hepatic steatosis in humans. *Cell Metab.* (2018) 27:559–571e5. 10.1016/j.cmet.2018.01.005 29456073 PMC6706084

[105] Mailing LJ Allen JM Buford TW Fields CJ Woods JA. Exercise and the gut microbiome: a review of the evidence, potential mechanisms, and implications for human health. *Exerc Sport Sci Rev.* (2019) 47:75–85. 30883471 10.1249/JES.0000000000000183

[106] Calabrese FM Disciglio V Franco I Sorino P Bonfiglio C Bianco A A low glycemic index mediterranean diet combined with aerobic physical activity rearranges the gut microbiota signature in NAFLD patients. *Nutrients.* (2022) 14:1773. 10.3390/nu14091773 35565740 PMC9101735

[107] Cheng R Wang L Le S Yang Y Zhao C Zhang X A randomized controlled trial for response of microbiome network to exercise and diet intervention in patients with nonalcoholic fatty liver disease. *Nat Commun.* (2022) 13:2555. 10.1038/s41467-022-29968-0 35538056 PMC9091228

[108] Sharpton SR Schnabl B Knight R Loomba R. Current concepts, opportunities, and challenges of gut microbiome-based personalized medicine in nonalcoholic fatty liver disease. *Cell Metab.* (2021) 33:21–32. 10.1016/j.cmet.2020.11.010 33296678 PMC8414992

[109] López-Salazar V Tapia MS Tobón-Cornejo S Díaz D Alemán-Escondrillas G Granados-Portillo O Consumption of soybean or olive oil at recommended concentrations increased the intestinal microbiota diversity and insulin sensitivity and prevented fatty liver compared to the effects of coconut oil. *J Nutr Biochem.* (2021) 94:108751. 33915261 10.1016/j.jnutbio.2021.108751

